# Mycobacterial RNA polymerase forms unstable open promoter complexes that are stabilized by CarD

**DOI:** 10.1093/nar/gku1231

**Published:** 2014-12-15

**Authors:** Elizabeth Davis, James Chen, Katherine Leon, Seth A. Darst, Elizabeth A. Campbell

**Affiliations:** Laboratory of Molecular Biophysics, The Rockefeller University, 1230 York Avenue, New York, NY 10065, USA

## Abstract

*Escherichia coli* has served as the archetypal organism on which the overwhelming majority of biochemical characterizations of bacterial RNA polymerase (RNAP) have been focused; the properties of *E. coli* RNAP have been accepted as generally representative for all bacterial RNAPs. Here, we directly compare the initiation properties of a mycobacterial transcription system with *E. coli* RNAP on two different promoters. The detailed characterizations include abortive transcription assays, RNAP/promoter complex stability assays and DNAse I and KMnO_4_ footprinting. Based on footprinting, we find that promoter complexes formed by *E. coli* and mycobacterial RNAPs use very similar protein/DNA interactions and generate the same transcription bubbles. However, we find that the open promoter complexes formed by *E. coli* RNAP on the two promoters tested are highly stable and essentially irreversible (with lifetimes much greater than 1 h), while the open promoter complexes on the same two promoters formed by mycobacterial RNAP are very unstable (lifetimes of about 2 min or less) and readily reversible. We show here that CarD, an essential mycobacterial transcription activator that is not found in *E. coli*, stabilizes the mycobacterial RNAP/open promoter complexes considerably by preventing transcription bubble collapse.

## INTRODUCTION

Tuberculosis, caused by infection with the bacterium *Mycobacterium tuberculosis* (*Mtb*), continues to pose a major health problem, particularly due to the increase in multidrug resistant strains (WHO Global tuberculosis report 2013 http://www.who.int/tb/publications/global_report/en/). RNA polymerase (RNAP), the enzyme responsible for all transcription in bacteria, is the target for the antibiotic rifampicin, a first line therapeutic treatment for tuberculosis (1), and is thus an attractive target for the development of new drugs.

The catalytic core of the bacterial RNAP, comprising five subunits (α_2_, β, β′ and ω), is competent for RNA synthesis (2). Promoter-specific transcription initiation, however, requires a promoter specificity factor, σ, which binds the core to form the holoenzyme (3,4). *Escherichia coli* (*Eco*) has served as the archetypal organism on which the overwhelming majority of biochemical characterizations of bacterial RNAP have been focused. The properties of *Eco* RNAP have been accepted as generally representative for all bacterial RNAPs.

The availability of high-resolution X-ray crystal structures of *Thermus aquaticus* (*Taq*) and *Thermus thermophilus (Tth)* RNAPs (5–7) has prompted biochemical characterization of these enzymes (8–13). Studies of transcription initiation by RNAPs from gram-positive organisms are sparse but include characterizations of RNAPs purified endogenously from *Bacillus subtilis* (14), *M. smegmatis* (15) and *Mtb* (16).

CarD [also called CdnL in *Myxococcus xanthus*; (17)] was identified as a direct RNAP binding protein that is an essential regulator of ribosomal RNA (rRNA) transcription in *Mtb* (18). CarD, although widely distributed across many eubacteria phyla, is not found in *Eco* (18,19). Loss of CarD is lethal for *Mtb* in culture and during infection of mice. Depletion of CarD results in sensitivity to killing by oxidative stress, starvation, DNA damage and changes in the mRNA levels of hundreds of genes. A combination of *in vivo* and *in vitro* approaches established that CarD is a global regulator that activates transcription by stimulating the formation of the RNAP/open promoter complex (19). The X-ray crystal structure of *Tth* CarD, combined with detailed structural and functional analyses, led to the proposal that CarD functions by forming protein/protein and protein/DNA interactions with a DNA structure uniquely presented by the open promoter complex (RPo)—the splayed minor groove at the double-/single-stranded DNA junction at the upstream edge of the transcription bubble.

Here, we directly compare the initiation properties of a mycobacterial transcription system with *Eco* RNAP on two different promoters. The detailed characterizations include abortive transcription assays, RPo stability assays and DNAse I and KMnO_4_ footprinting. We find that the tested open promoter complexes formed by *Eco* RNAP are highly stable and essentially irreversible, while the same open promoter complexes formed by mycobacterial RNAP are very unstable and readily reversible (in equilibrium with RNAP and free promoter or other intermediates on the promoter melting pathway). The transcription activator CarD stabilizes the mycobacterial RNAP/open promoter complexes considerably by preventing collapse of the transcription bubble, thereby compensating for the enzyme's relatively feeble activity on a*Mtb* rRNA promoter.

## MATERIALS AND METHODS

### Protein purification

*Eco* core RNAP was overexpressed and purified from *Eco* BL21(DE3) cells co-transformed with pGEMABC (encoding *Eco* RNAP *rpoA*, *rpoB* and *rpoC*; Addgene plasmid 45398) and pACYCDuet-1_Ec_rpoZ (encoding *rpoZ*) as described (20). *Eco* σ^70^ was overexpressed and purified as described (21). *M. bovis* (*Mbo*) core RNAP and σ^A^ were overexpressed and purified using methods modified from Czyz *et al.* (22). Briefly, the *Mbo* core RNAP subunits were co-overexpressed in *Eco* pRARE2 (Novagen) cells overnight at room temperature for ∼16 h after induction with 0.1 mM isopropyl-beta-D-thiogalactopyranoside (IPTG). Cell pellets were lysed with a continuous flow French press (Avestin) and the clarified cell lysate was treated by polyethyleneimine (PEI) precipitation to remove nucleic acids. Proteins eluted from the PEI pellet were then purified by Ni^2+^—affinity chromatography and the eluted sample concentrated by centrifugal filtration (VivaSpin) and further purified by size exclusion chromatography. Buffers and detailed methods are as described in detail in Twist *et al.* (23). *Mbo/Mtb* σ^A^ was overexpressed in *Eco* pRARE2 cells and purified as previously described (22). *Mbo/Mtb* CarD was overexpressed from *Eco* BL21(DE3) as previously described for *Tth* CarD (19).

### Transcription assays

An AC50 [also called -35con; (24)] promoter DNA fragment (−152 to +72) was polymerase chain reaction (PCR) amplified from plasmid pAC50 (22). Promoter DNA (−60 to +15) of *rrnA*P3 from *Mtb* (25) was synthesized (GenScript) and placed into the pUC57 plasmid to generate pUC57-AP3. Fragment −86 to +70 of pUC57-AP3 was also PCR amplified. Both promoter DNA fragments were subsequently subjected to agarose electrophoresis and gel purified (Qiagen). These promoter fragments (AC50 and AP3) served as templates for all biochemical assays except where described otherwise. Artificial transcription bubble and double-stranded templates of AC50 (−60 to +20) were synthesized and gel purified (IDT). Purified oligos were then annealed and used as templates for transcription as described in Figure 7.

**Figure 1.**
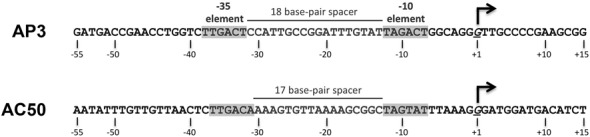
Sequences of the *Mtb* AP3 [*Mtb rrnA*–P3; (25)] and AC50 [*-35con* of (24)] promoters.

**Figure 2.**
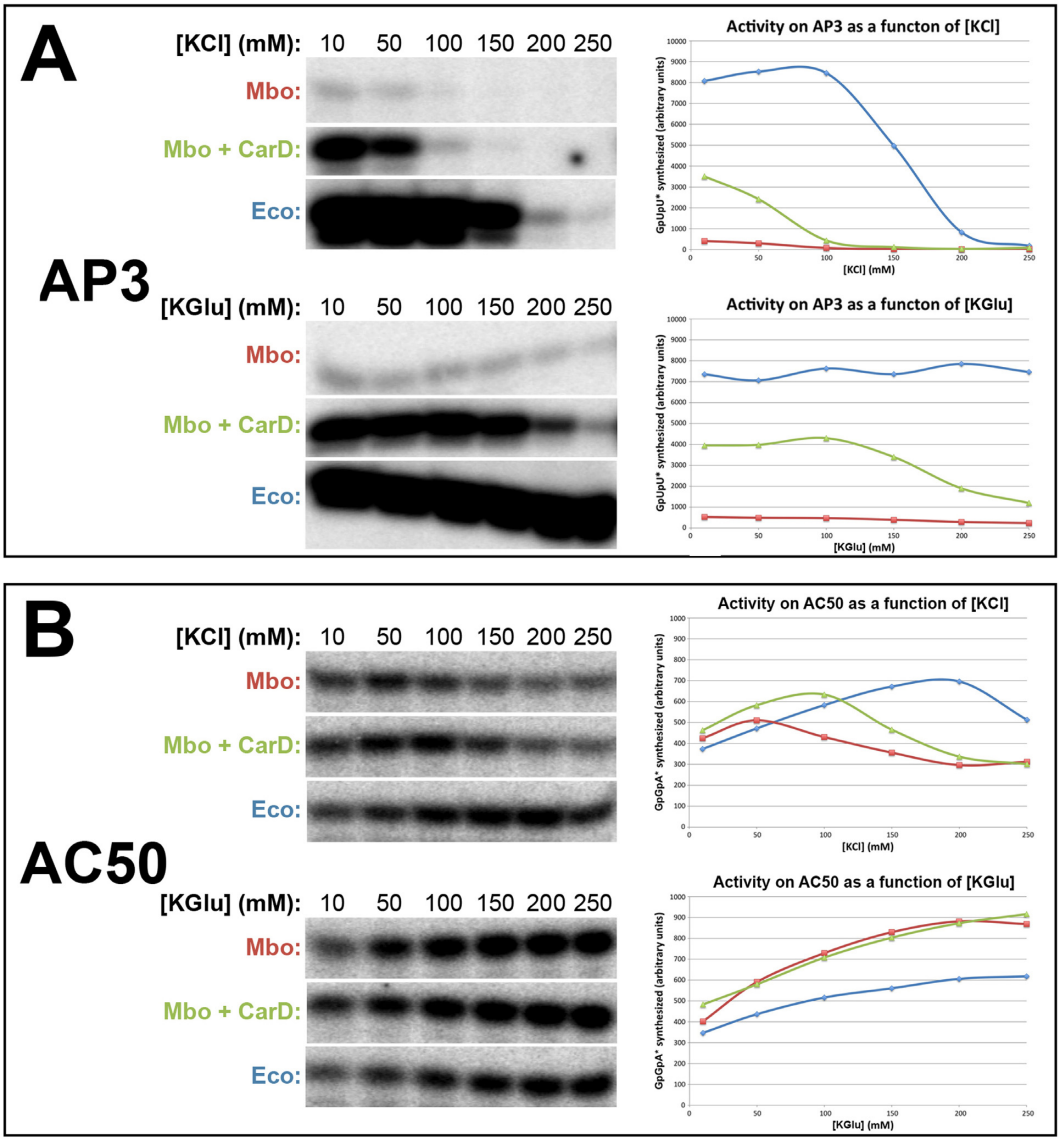
Dependence of *Mbo* and *Eco* RNAP transcription activity on salt concentration for both the AP3 and AC50 promoters. Single round abortive initiation assays measured GpUpU (AP3) or GpGpA (AC50) production. On the left, [^32^P]-labeled abortive transcript production was monitored by polyacrylamide gel electrophoresis and autoradiography. On the right, transcript production was quantified by phosphorimagery and plotted versus [KCl] (top) or [KGlu] (bottom) concentration (10–250 mM). On the plots, *Mbo* RNAP (alone) is shown in red, *Mbo* RNAP + CarD in green, *Eco* RNAP in blue. (**A**) AP3 promoter. (**B**) AC50 promoter.

**Figure 3.**
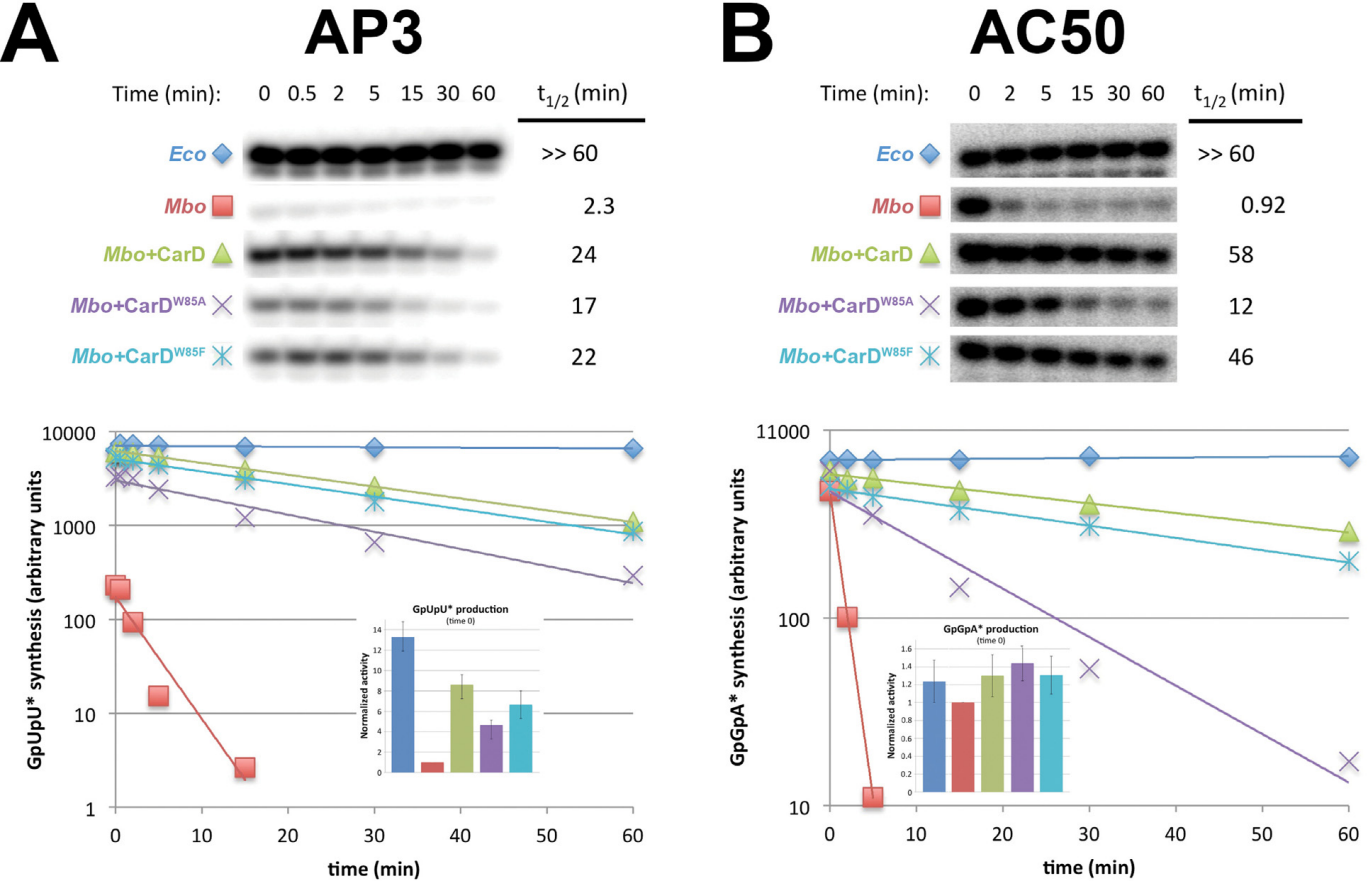
Lifetimes of promoter complexes measured by abortive transcription. On the top, [^32^P]-labeled abortive transcript production at times after addition of a large excess of competitor promoter DNA trap was monitored by polyacrylamide gel electophoresis and autoradiography. On the bottom, transcript production was quantified by phosphorimagery and plotted. The lines indicate single-exponential decay curves fit to the data points. The decay half-lives (t_1/2_) calculated from the fits are shown to the right of the gel images. The insets show histograms denoting transcription activity at time 0 (before incubation with competitor trap DNA). (**A**) AP3 promoter: assays were performed in transcription buffer (see Materials and Methods) with 10 mM KGlu. (**B**) AC50 promoter: assays were performed in transcription buffer (see Materials and Methods) with 150 mM KGlu.

**Figure 4.**
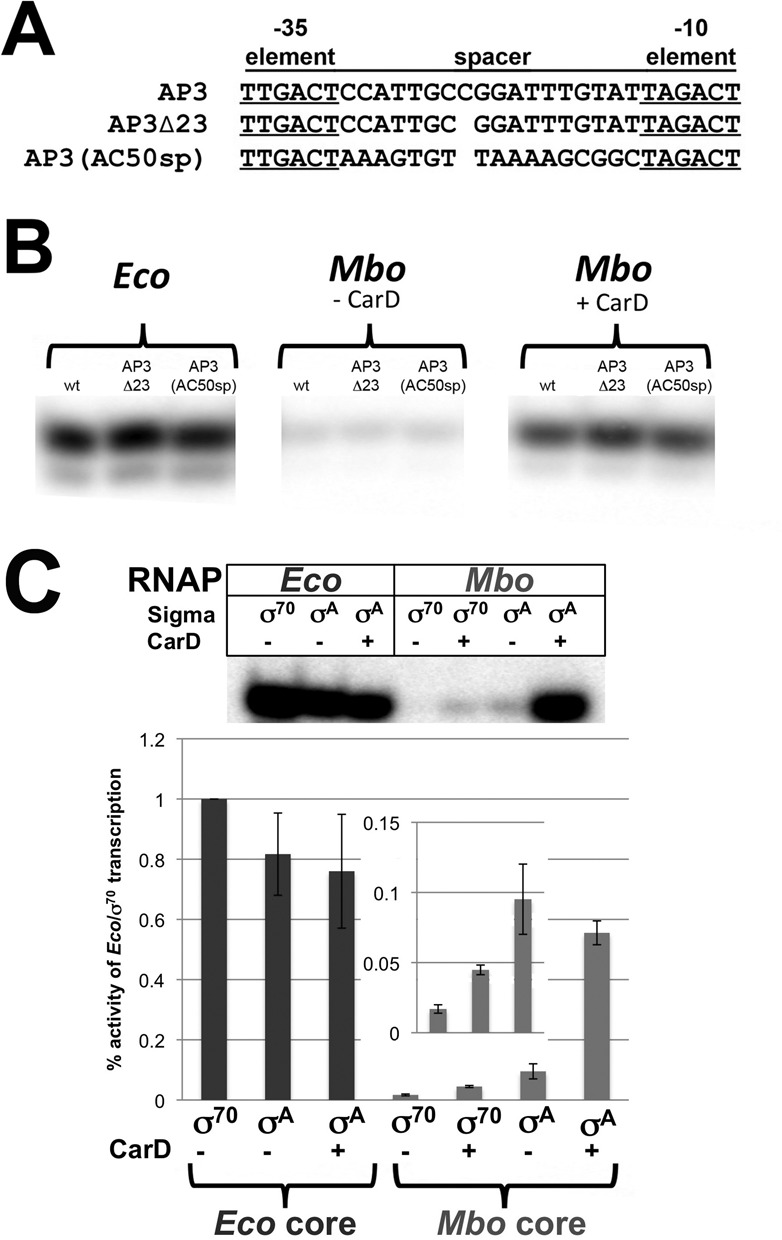
Weak activity of *Mbo* RNAP on the AP3 promoter is not due to the suboptimal 18-bp −10/-35 spacer of AP3 nor *Mbo* σ^A^. (**A**) Sequences of AP3 (18-bp −10/-35 spacer, top) and spacer mutant promoters. AP3Δ23 has a deletion of the −23 bp, giving AP3Δ23 a 17-bp −10/-35 spacer. AP3(AC50sp) has the optimal 17-bp spacer of AC50 (blue) swapped for the AP3 spacer. (**B**) Single round abortive initiation activity of RNAPs on wt AP3, AP3Δ23 and AP3(AC50sp) was determined in transcription buffer as described (Figure 2 and Materials and Methods) with 10 mM KGlu. Gels show transcription initiation products (GpUpU* synthesis). (**C**) Single round abortive initiation assays were performed as described in (B) with hybrid holoenzymes. *Eco* core RNAP was mixed with either *Eco* σ^70^ or *Mbo* σ^A^ and assayed for activity on the AP3 promoter. The reverse experiment was also performed with *Mbo* core RNAP mixed with *Eco* σ^70^ or *Mbo* σ^A^. CarD was also tested for effects on transcription where indicated. Graphs below represent relative activities of hybrid holoenzymes normalized to *Eco*-core/σ^70^ holoenzyme. The inset shows a magnification of *Mbo*-core/*Eco* σ^70^, *Mbo*-core/*Eco* σ^70^ + CarD and *Mbo*-core/*Mbo* σ^A^ to better visualize the weak activity (<0.1% that of *Eco*-core/σ^70^).

**Figure 5.**
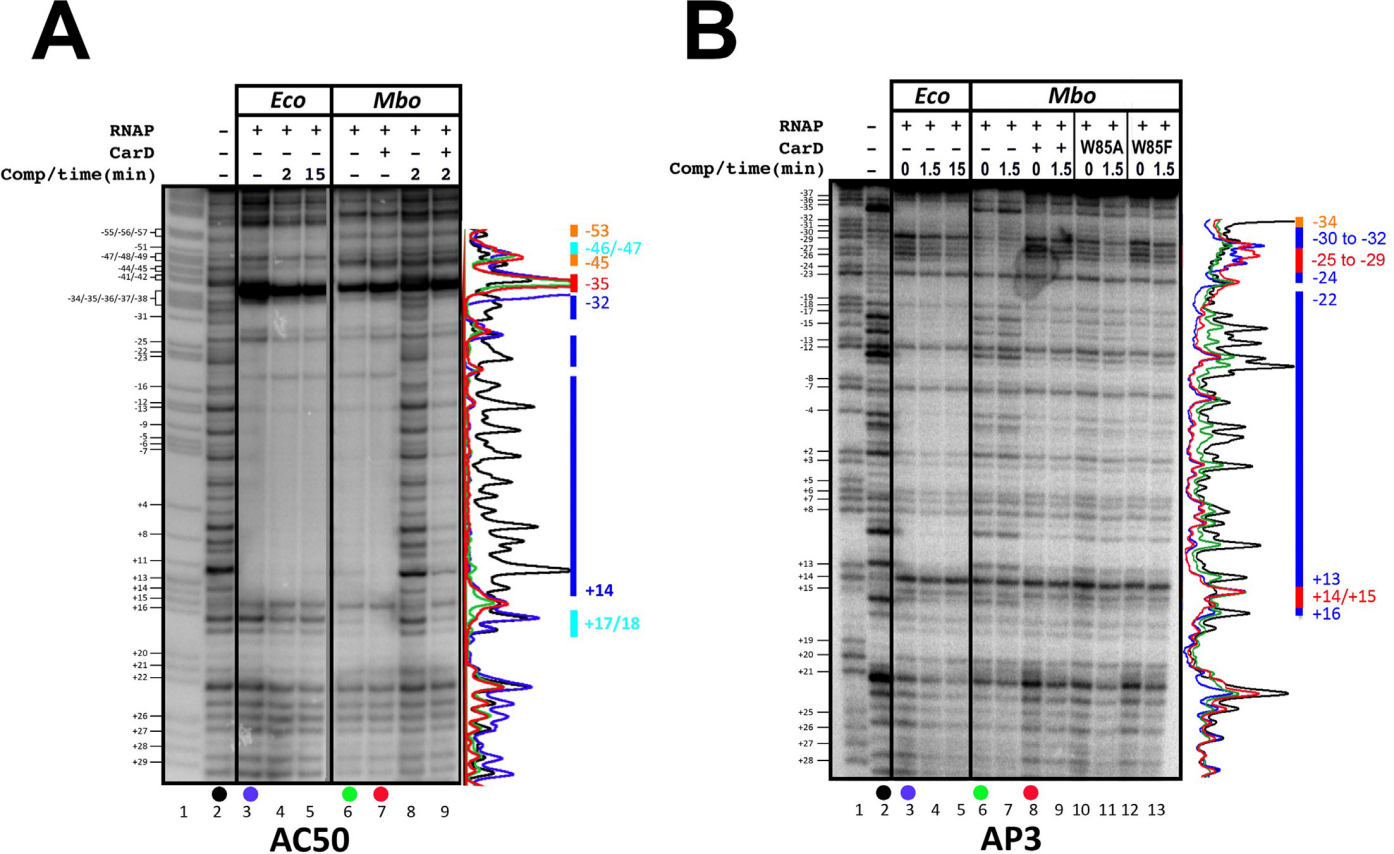
DNAse I footprints (template strand) of *Eco* and *Mbo* (±CarD) RNAPs on the AP3 and AC50 promoters. In each panel (A and B), lane 1 shows the AG sequencing ladder (assignments shown on the left), lane 2 shows DNAse I cleavage in the absence of any proteins. DNAse I footprints are shown without competitor trap, and with competitor trap incubation prior to cleavage (times as indicated). The colored bars on the right denote the footprint characteristics (blue, DNAse I protection for both *Eco* and *Mbo* RNAPs; red, DNAse I hypersensitivity for both RNAPs; orange, protection by *Eco* RNAP but not *Mbo* RNAP; cyan, protection by *Mbo* RNAP but not *Eco* RNAP). Densitometric traces provided on the right illustrate the protection profiles. Colors of each trace correspond to samples indicated by the colored dots below the gel lanes. (**A**) AP3 promoter: protection by *Mbo* RNAP alone is not as apparent as with CarD, therefore, the blue bars only represent protection by *Eco* RNAP and *Mbo* RNAP + CarD. (**B**) AC50 promoter.

**Figure 6.**
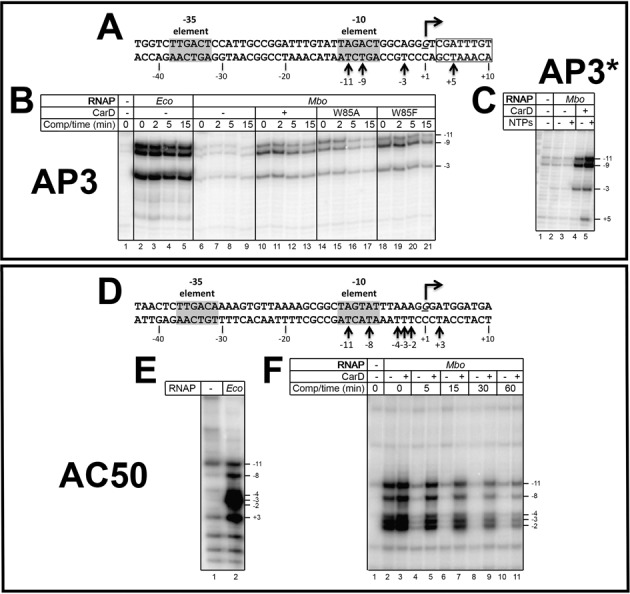
KMnO_4_ footprints (template strand) of *Eco* and *Mbo* (±CarD) RNAPs on the AP3 and AC50 promoters. (**A**) Sequence of the AP3 promoter, except the altered sequence (+3 and downstream) of AP3* is boxed (gives rise to the template-strand T at +5 which is absent in AP3, see Figure 1). Template strand (bottom) thymidines rendered KMnO_4_ reactive by RNAP are denoted. (**B**) KMnO_4_ footprints. Lane 1, no protein added. (**C**) Effect of adding initiating NTPs (GpU ribonucleotide dimer and CTP) on the KMnO_4_ footprint of *Mbo* RNAP on the AP3* promoter. (**D**) Sequence of the AC50 promoter. Template strand (bottom) thymidines rendered KMnO_4_ reactive by RNAP are denoted. (**E**) KMnO_4_ footprint of *Eco* RNAP on the AC50 promoter. (**F**) KMnO_4_ footprints of *Mbo* RNAP (±CarD as indicated) on the AC50 promoter.

**Figure 7.**
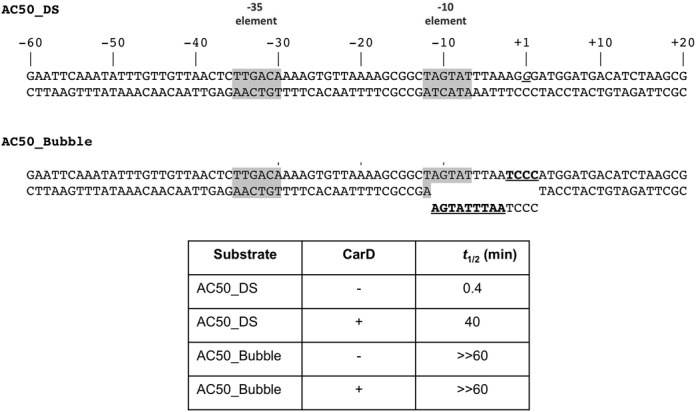
A non-complementary transcription bubble rescues short half-life of *Mbo* RPo on the AC50 promoter and renders CarD redundant. The AC50 double-stranded promoter (from −60 to +20) was synthesized and used as a template for abortive transcription assays (AC50-DS). In AC50-bubble, non-complementary mismatches (underlined) were introduced from −11 to +2, generating a non-collapsible transcription bubble (AC50_Bubble). Half-life assays were performed and RPo half-lives calculated as described in Figure 3B.

Proteins used for the *in vitro* transcription assays were diluted into 1× transcription buffer [10 mM Tris-HCl, pH 8.0, 10–150 mM KGlu (unless otherwise noted), 1 mM MgCl_2_, 0.1 mM DTT, 50 μg/ml bovine serum albumin]. Reactions (20 μl) were carried out in a 37°C water bath with proteins using the following protocol: core RNAP (200 nM) and σ^A^ (1 μM) were combined and incubated at 37°C for 5 min to form holoenzyme. CarD (2 μM, when used) was then added to holoenzyme and incubated for an additional 5 min. Next, promoter DNA (10 nM) was added and RP_O_ was allowed to form for 15 min at 37°C. Abortive transcription was initiated by the addition of an NTP mix containing the initiating dinucleotide (250 μM, GpU for AP3, GpG for AC50; TriLINK), the next NTP (α-^32^P labeled, UTP for AP3, ATP for AC50; 1.25 μCi, with 50 μM of the same unlabeled NTP) and FC-bubble competitor DNA (2 μM, see below). After 10 min, transcription was quenched by the addition of 2× stop buffer (8 M Urea, 0.5× TBE, 0.05% Bromophenol blue, 0.05% Xylene cyanol). Reactions were heated at 95°C for 1 min and immediately loaded on a 23% polyacrylamide gel (19:1 acrylamide:bis-acrylamide). Abortive products were visualized by exposing the gel on a phosphorimager plate overnight and digitized using a Typhoon phosphorimager. Data were quantified using Image J (26).

### DNase I footprinting

Promoter DNAs with 5′-end-labeled template strand was prepared by PCR amplification using a 5′-[^32^P]-end-labeled PCR primer. The resulting PCR products were then gel purified as described above. DNaseI (New England Biolabs) was diluted to 500 U/μl and kept on ice. Reactions (25 μl) were carried out in a 37°C water bath and in 1× transcription buffer. Core RNAP (400 nM) and σ^A^ (2 μM) were incubated for 5 min to form holoenzyme. CarD (4 μM), when used, was added to the holoenzyme and incubated for another 5 min, followed by the addition of the ^32^P-labeled promoter DNA (200 fmol). Formation of RPo was allowed to proceed for 15 min and competitor added for times indicated (Figure 5). DNase I (500 U) was then added to the mixture and the reaction incubated for an additional 2 min. The reactions were quenched by the addition of 100 μl of 0.5 M phenol, 75 μl of sodium acetate (0.3 M) and ethylenediaminetetraacetic acid (10 mM final). The DNA was recovered in the aqueous layer, ethanol precipitated and washed. The air-dried pellet was resuspended in 2× loading buffer, heated at 95°C for 1 min before being immediately loaded on an 8% polyacrylamide (19:1 acrylamide:bis-acrylamide) 8M urea gel. The gel was visualized as described above.

### KMnO_4_ footprinting

Open complexes were formed on 5′-[^32^P]-labeled (template strand) promoter DNA as described above for DNAse I footprinting. Potassium permanganate (KMnO_4_) was added to a final concentration of 2 mM, incubated for 2 min, then the reactions were quenched by the addition of 25 μl of stop buffer (1 M β-mercaptoethanol, 1.5 M sodium acetate). The DNA was precipitated with 200 μl EtOH, pelleted, then washed with 100% EtOH to remove all traces of KMnO_4_. The DNA pellet was then resuspended in 100 μl of piperidine (1 M) and incubated at 90°C for 30 min to cleave the DNA at modified thymine residues. After cleavage, the DNA was precipitated with 100% EtOH, pelleted, washed, air-dried, re-suspended in 2× loading buffer and subjected to electrophoresis and visualized as described for the DNase I assays.

## RESULTS

### Mycobacterial transcription system

Recombinant *Mbo* core RNAP was generated by co-overexpression and *in vivo* assembly of the RNAP subunits (α, β, β′, ω) in host *Eco* cells and purified to homogeneity using a revised purification procedure (22) (Supplementary Figure S1). The *Mbo* housekeeping promoter specificity factor, σ^A^, was also overexpressed in *Eco* and purified as described (22). The *Mbo* holoenzyme is identical to that of the pathogen *Mtb* with the exception of one amino acid (*Mbo* β’P69 is R69 in *Mtb*). CarD is identical between *Mbo* and *Mtb*. Our analyses focused on two promoters, *Mtb rrnA*-P3 (*Mtb* AP3), a promoter of the *Mtb rrnA* operon (25), and AC50, based on *-35con* of Gaal *et al.* (24) (Figure 1). The AP3 promoter has a nearly consensus −35 element but a non-optimal 18 base-pair (bp) spacer between the −10 and −35 elements (Figure 1). The AC50 promoter harbors an optimized −35 element and an optimal 17 bp −10/−35 spacer. On AC50 at saturating concentration of RNAP, *Mbo* RNAP holoenzyme showed similar levels of transcription activity as *Eco* RNAP, indicating comparable activity ((22); Figures 2 and 3).

### Salt sensitivity of *Mbo* RNAP promoter complexes

Single round abortive initiation assays were used to compare the activity of *Eco* and *Mbo* RNAPs as a function of [KCl] and [K-glutamate] ([KGlu]) on both the AP3 and AC50 promoters. Although KCl or NaCl are typically used in *in vitro* transcription studies, in *Eco* cells, [Cl^−^] is always very low. The primary anion is glutamate, which varies dramatically in concentration (30–260 mM) depending on the osmolarity of the surrounding medium (27). Even higher levels of intracellular glutamate have been estimated for *Mtb* (28). Previous studies found that moderate concentrations of KGlu significantly stabilized *Eco* RNAP interactions with some promoters compared to KCl or NaCl (29). In particular, transcription from the *Eco rrnB* promoter was particularly sensitive to Cl^−^ but tolerated high concentrations of KGlu. However, the effect was promoter specific. For instance, the *lac UV5* promoter maintained the same activity in both low and high concentrations of KCl and KGlu (30).

On the AP3 promoter, *Eco* RNAP showed high activity in KCl up to 100 mM which then dropped off rapidly at increasing [KCl] (Figure 2A; half-maximal activity at ∼160 mM [KCl]). By contrast, activity of *Mbo* RNAP was more than an order of magnitude less and was essentially completely absent at 100 mM KCl and above (half-maximal activity at ∼60 mM [KCl]). Saturating amounts of CarD boosted *Mbo* RNAP transcription almost 9-fold but did not alter the KCl-sensitivity (half-maximal activity at ∼60 mM [KCl]).

In low [KGlu] (≤50 mM) on AP3, both *Eco* and *Mbo* (±CarD) RNAPs showed very similar activites as in low [KCl]. However, both RNAPs were much less sensitive to increasing [KGlu]. *Eco* RNAP activity was completely resistant to increasing [KGlu] up to the highest [KGlu] tested, 250 mM (Figure 2A). *Mbo* RNAP, both with and without CarD, was also more resistant to increasing [KGlu] (half-maximal activity at ∼200 mM [KGlu]).

Like on the AP3 promoter, the activity of *Eco* RNAP on AC50 was much less sensitive to increasing [KCl] (≥150 mM) than *Mbo* RNAP (with or without CarD; Figure 2B). The similarity of each enzyme's activity on AP3 as a function of [salt] ended there, however. Unlike on the AP3 promoter, at low [KCl] (≤50 mM), both *Eco* and *Mbo* activities were similar to each other, and *Mbo* activity was not dependent on CarD. In KGlu, the activities of both *Eco* and *Mbo* RNAPs increased with increasing [KGlu] (up to 250 mM). For all subsequent assays (both *Eco* and *Mbo* RNAPs on both AP3 and AC50 promoters), transcription conditions used are 10 mM (for AP3) or 150 mM (for AC50) [Kglu], as noted.

### *Mbo* RNAP promoter open complexes are extremely unstable compared to *Eco* RNAP, a phenotype partially rescued by CarD

We tested the lifetime of competitor-resistant RNAP/promoter complexes using the abortive initiation assay in KGlu. For these and other assays where promoter complexes were challenged with competitor, we chose not to use heparin, which actively dissociates RNAP from promoters (31). Instead, we designed a competitive promoter trap comprising the optimized full-con promoter sequence (24) but also with a non-complementary transcription bubble to afford rapid and irreversible RNAP binding (FC-bubble, Supplementary Figure S2A). Control experiments demonstrated that the FC-bubble is an extremely effective competitor (Supplementary Figure S2B).

With *Mbo* RNAP on both promoters, we observed biphasic decay with a rapidly decaying component (t_1/2_ ∼2 min) with a decay rate strongly dependent on the presence of CarD, and a very slow component (t_1/2_ ∼5 h) that did not seem to depend on CarD (Supplementary Figure S3). On AC50, the slow-decaying component could be essentially eliminated by increasing [KGlu]. The experiments shown in Figure 3B were obtained in 150 mM [KGlu], giving rise to single-exponential decay kinetics. For AP3, *Mbo* RNAP transcription activity in the absence of CarD was extremely weak and only decreased with higher [KGlu] (Figure 2A). We therefore chose to perform the AP3 promoter lifetime experiments at 10 mM KGlu and removed the slow-decaying component from the data shown in Figure 3A (see Supplementary Figure S3). Under these conditions, the slow-decaying component accounted for less than 10% of the transcription activity of *Mbo* RNAP with CarD, and ∼50% of the (extremely weak) activity of *Mbo* RNAP without CarD (Supplementary Figure S3).

On the AP3 promoter, *Eco* RNAP showed high transcription activity that was essentially completely competitor resistant over the 60 min assay time (Figure 3A). By contrast, *Mbo* RNAP showed very weak transcription that decayed rapidly (t_1/2_ = 2.3 min). The presence of saturating amounts of CarD stimulated *Mbo* RNAP transcription by almost an order of magnitude (consistent with previous results) to nearly the same level as *Eco* RNAP (at time 0). CarD also dramatically stabilized the *Mbo* RNAP promoter complexes, increasing the t_1/2_ by more than 10-fold (Figure 3A).

Substitution of conserved CarD W85 to A (CarD^W85A^) showed that this residue is critical for optimal transcription of the rRNA promoters in both *Tth* and *Mtb* (19). We tested both CarD^W85A^ and a more conservatively substituted mutant, CarD^W85F^, in our transcription assays. Consistent with previous results, CarD^W85A^ showed a roughly 2-fold reduction in transcription activation (54% of the transcription activity compared to wt-CarD at time 0) but was still able to stabilize the *Mbo* RNAP promoter complexes to nearly the same level as wt-CarD (Figure 3A). CarD^W85F^ showed near wild-type activity when compared to CarD^W85A^ (77% versus 54% shown by CarD^W85A^ of the transcription activity compared to wt-CarD at time 0).

On the AC50 promoter, *Eco* RNAP also showed high transcription activity that was essentially completely competitor resistant over the 60 min assay time (Figure 3B). *Mbo* RNAP also showed strong transcription activity (69% of *Eco* RNAP activity), but this activity decayed extremely rapidly upon competitor challenge (t_1/2_ = 0.92 min). Saturating amounts of CarD had only a slight effect on *Mbo* RNAP transcription at time 0 (1.3-fold activation) but, like on AP3, dramatically stabilized the complexes to dissociation (63-fold increase in t_1/2_ compared to no CarD). The CarD^W85A^ mutant had little effect on *Mbo* RNAP transcription at time 0, and stabilized the promoter complexes at an intermediate level (13-fold increase in t_1/2_ compared to no CarD). As with the AP3 promoter, CarD^W85F^ was able to stabilize the open complex on AC50 at levels similar to wt-CarD (Figure 3B).

In summary, under these assay conditions, *Eco* RNAP formed exceedingly stable promoter complexes on both the nearly optimal AC50 and the non-optimal AP3 promoters (t_1/2_'s >> 60 min), while promoter complexes of *Mbo* RNAP were highly unstable (t_1/2_ = 0.92 and 2.3 min, respectively). CarD had two major effects on *Mbo* RNAP initiation, to activate the overall level of transcription (nearly 9-fold on AP3, but only slightly on AC50) and to stabilize the otherwise highly unstable *Mbo* RNAP promoter complexes (on both promoters). The CarD^W85A^ substitution caused a partial loss of transcription activation (on AP3) activity as well as a partial defect in promoter complex stabilization (on both promoters) while CarD^W85F^ showed stabilization similar to that of wt-CarD.

### The suboptimal 18 bp −10/−35 spacer is not the origin of the weak transcription activity on the AP3 promoter

The *Mtb* AP3 promoter has a non-optimal 18-bp −10/−35 spacer (Figure 1). Nevertheless, *Eco* RNAP transcribes the *A*P3 promoter well and forms very stable promoter complexes (Figure 3A). On the other hand, *Mbo* RNAP shows very weak activity in the absence of CarD that is very sensitive to [KCl] (Figure 2A), and forms unstable promoter complexes (Figure 3A). To test whether *Mbo* RNAP was particularly sensitive to the non-optimal 18-bp spacer, we examined two engineered AP3 derivatives with optimal 17-bp spacers. In AP3Δ23, we generated a 17-bp spacer by deleting the bp at −23 (Figure 4A). In AP3(AC50sp), we swapped the 18-bp AP3 spacer for the 17-bp AC50 spacer (Figure 4A). Single round abortive initiation assays were performed on these promoters using RNAP from *Eco* and *Mtb* (±CarD) (Figure 4B). None of the promoter alterations had a significant effect on the efficiency of transcription by either RNAP. Thus, the poor transcription of the AP3 promoter by *Mbo* RNAP (in the absence of CarD) is not due to the non-optimal, 18-bp spacer.

### *Mbo* σ^A^ is not responsible for the weak transcription activity on the AP3 promoter

*Mbo* **σ^A^** is very similar to *Eco* **σ^70^** and the residues contacting the −10 and −35 promoter regions are highly conserved (32,33). In contrast, the non-conserved regions (NCR), inserted within domain 2 of housekeeping **σ** factors (34), share virtually no homology. Therefore, we tested whether *Mbo* σ^A^ was the cause of the instability and weak activity of the *Mbo* holoenzyme by comparing the transcriptional activity of *Eco* core RNAP with *Eco* **σ^70^** (*Eco*-core/**σ^70^)** to *Eco* core RNAP with *Mbo* **σ^A^** (*Eco*-core/**σ^A^)** on the AP3 promoter. *Mbo* **σ^A^** was able to direct transcription of AP3 by *Eco* core RNAP almost as well as **σ^70^**, suggesting that the weak activity of *Mbo* holoenzyme does not originate from **σ^A^** (Figure 4C). As expected, CarD was unable to activate *Eco*-core/**σ^70^** (data not shown) nor *Eco*-core/**σ^A^** holoenzymes (we have no evidence that CarD interacts with *Eco* RNAP).

Previous studies found that interaction between *Eco* **σ^70^** and *Mbo* core RNAP was not detectable by native gel electrophoresis (22). Therefore, as expected, *Eco* **σ^70^** directed only very weak transcription of AP3 by the *Mbo* core RNAP (Figure 4C). One possible explanation for the weak *Eco* σ^70^/*Mbo* RNAP interaction is that the relatively large NCR of *Eco* **σ^70^** (247 residues, compared to the 32 residue *Mbo* σ^A^ NCR) could clash with lineage-specific insert 2 of the mycobacterial RNAP β′ subunit (35), a ∼130-residue insert located near the σ NCR that is not present in *Eco*. CarD very weakly activates *Mbo*-core RNAP/Eco σ^70^ (Figure 4C).

### DNase I footprinting

Occupancy of the promoter by RNAP protects the DNA from DNAse I cleavage, usually over a range from about −55 to +20 (36–39). DNAse I binds DNA in the minor groove, widening it and bending the DNA away from itself toward the major groove (40). Thus, RNAP-mediated DNA distortion resulting in exposed, widened minor grooves can also give rise to sites of DNAse I hypersensitivity. We examined promoter complexes of *Eco* and *Mbo* RNAPs on both the AP3 and AC50 promoters by DNase I footprinting (Figure 5).

On the AP3 promoter, *Eco* RNAP produced a strong DNAse I footprint, protecting the promoter DNA completely from ∼−22 to +13. Additional regions of protection and hypersensitivity extended upstream to −34 (Figure 5A, lanes 3–5). By contrast, *Mbo* RNAP (without CarD) failed to protect most of the DNA from DNAse I cleavage. Evidence of *Mbo* RNAP interaction with the DNA was apparent through the presence of some hypersensitive sites, such as at −34 and +14/+15 (Figure 5A, lanes 6–7). Addition of CarD conferred partial DNA protection (Figure 5A, lanes 8–9). The CarD^W85^ substitutions (W85A, W85F) produced footprints similar to wild-type CarD (Figure 5A, lanes 10–13). The DNAse I footprint due to *Eco* RNAP binding was resistant to competitor (Figure 5A, lanes 4–5), as was *Mbo* RNAP in the presence of CarD (Figure 5A, lanes 9, 11, 13).

On the AC50 promoter, *Eco* and *Mbo* RNAPs produced strong DNAse I footprints that were very similar to each other. Some minor differences in the footprints were observed upstream of the −35 element (−45 to −53), and the *Mbo* RNAP footprint extended slightly further downstream (+17/+18) than the *Eco* RNAP footprint (Figure 5B, lanes 3–5 compared to lanes 6–7). Addition of CarD had no effect whatsoever on the *Mbo* RNAP footprint (Figure 5B, compare lanes 6 and 7). This is not surprising since the region of DNA expected to be contacted by CarD [just upstream of the −10 element; (19)] is already completely protected from DNAse I cleavage by *Mbo* RNAP alone. The *Mbo* RNAP footprint was very sensitive to the presence of competitor DNA. CarD substantially stabilized the *Mbo* RNAP complex to competitor (Figure 5B, lanes 8–9).

### KMnO_4_ footprinting

KMnO_4_ reacts with unstacked thymine (T) bases, and the modified T's can be subsequently detected by strand cleavage. This approach can thus be used to probe transcription bubble formation in RPo (39,41). We used KMnO_4_ footprinting (template strand) to examine the transcription bubble formed by *Eco* and *Mbo* RNAPs, and to examine the effect of CarD on *Mbo* RNAP transcription bubble formation.

The transcription bubble formed by *Eco* RNAP holoenzyme at promoters, as measured by KMnO_4_ footprinting, typically extends from about −11 to +2 (41). On the AP3 promoter, the transcription bubble formed by *Eco* RNAP results in strong KMnO_4_ reactivity of template strand T's at −11, −9 and −3, the only T's present within the typical transcription bubble range (Figure 6A, B, lane 2). *Mbo* RNAP, without CarD, formed a barely detectable transcription bubble (Figure 6B, lane 6). Transcription bubble formation by *Mbo* RNAP was stimulated dramatically by CarD (Figure 6B, lane 10). The extent of the transcription bubbles formed by *Mbo* and *Eco* RNAPs was identical, as far as could be determined.

Addition of NTPs has been shown to extend DNAse I protection of the *rrnB* P1 promoter downstream of the transcription bubble and to increase the intensity of KMnO_4_-reactive thymines (42). Therefore, we tested whether addition of NTPs would stimulate and possibly extend bubble formation on AP3 by *Mbo* RNAP. In order to detect if the bubble would be extended by the addition of NTPs, we used an alternative construct of AP3 (AP3*) where the sequence from +3 and downstream was altered, generating a template-strand T at +5 (Figure 6A). Addition of a dinucleotide primer (GpU) and CTP supported the abortive synthesis of GpUpC (Figure 2A) but did not enhance bubble formation by *Mbo* RNAP without CarD (Figure 6C, lanes 2 and 3). In the presence of CarD KMnO_4_ reactivity was slightly enhanced upon the addition of the initiating nucleotides. In addition, the bubble extended downstream to +5 (Figure 6C, lanes 4 and 5), most likely due to ‘scrunching’ during abortive synthesis (43,44).

The CarD^W85A^ mutant stimulated bubble formation somewhat less than wt-CarD (Figure 6B, compare lanes 10 and 14), and upon challenge with competitor, dissociated more rapidly. The CarD^W85F^ mutant stimulated bubble formation similar to wild-type CarD (Figure 6B, lanes 18–21).

On AC50, RPo formation by *Eco* RNAP induced KMnO_4_ reactivity at all template-strand T's between −11 and +3 (−11, −8, −4, −3, −2, +3; Figure 6D and E). *Mbo* RNAP without CarD induced a very similar footprint except the template strand T at +3 was relatively much less reactive (Figure 6F, lane 2). With CarD, KmnO_4_ reactivity was slightly stimulated, but the pattern of reactivity was unaltered (Figure 6F, lanes 2 and 3). The striking effect of CarD was seen after challenging the complexes with competitor, where the promoter complexes were dramatically stabilized over time by CarD (Figure 6F).

### CarD stabilizes RPo by preventing transcription bubble collapse

CarD stabilizes *Mbo* RNAP open complexes on the AC50 promoter template (spanning −152 to +72), increasing the half-life in the presence of competitor more than 60-fold (Figure 3B). We hypothesized that prevention of transcription bubble collapse (reannealing) could explain the dramatic effect of CarD on RPo stability. To test this hypothesis, we determined the effect of CarD on *Mbo* holoenzyme stability on a synthetic promoter template based on the AC50 sequence (spanning −60 to +20; Figure 7, AC50_DS) and compared it with the exact same synthetic template but with a non-complementary transcription bubble (from −11 to +2; Figure 7, AC50_Bubble) unable to collapse. The non-complementary bubble was generated by altering the template strand sequence from −11 to −3 [thus maintaining the −10 element and discriminator sequences on the non-template strand (33,45,46)] and the non-template strand from −2 to +2 (thus maintaining the same initially transcribed sequence). Half-life assays were performed as described for Figure 3. On the AC50_DS template, CarD increased the half-life 100-fold (Figure 7), consistent with our results on the full AC50 double-stranded template (Figure 3B). However, on the AC50_Bubble template, *Mbo* holoenzyme behaved much like *Eco* holoenzyme, with no detectable dissociation over the 60-min experiment (half-life >> 60 min), and addition of CarD had no effect. We conclude that the very short half-life of *Mbo* RPo on AC50 (Figure 3B) is due, at least in large part, to collapse of the transcription bubble, which generates the closed promoter complex (RPc) at the expense of RPo. RPc is in rapid equilibrium with RNAP and promoter DNA in solution, and in the presence of the full-con promoter trap competitor DNA, transcription competent RPo is rapidly depleted. The stabilization of *Mbo* RPo by CarD can be largely attributed to the inhibition of transcription bubble collapse by CarD.

## DISCUSSION

In this study, we used a mycobacterial transcription system (*Mbo* core RNAP, *Mbo*/*Mtb* σ^A^ and *Mbo*/*Mtb* CarD—essentially identical to a complete *Mtb* transcription system) that shows transcription activity on the optimized AC50 promoter (Figure 1) essentially equal to or better than the very well established *Eco* transcription system (Figure 2B). We thus propose that differences in behavior we observed between the *Mbo* and *Eco* RNAPs can be attributed to mechanistic differences in enzyme function and not due to suboptimal RNAP preparations or conditions.

Our studies revealed some similarities between the two RNAPs. These include:

- Both the *Mbo* and *Eco* RNAPs gave rise to nearly identical Dnase I footprints on each promoter (Figure 5), indicating similar physical protein/DNA interactions.
- The RNAPs gave rise to nearly identical KMnO_4_ footprints on each promoter (Figure 6), indicating very similar transcription bubbles in the open complexes.

Despite these similarities listed above, the mechanistic differences we have observed between the two RNAPs are profound, and include:

- Weak transcription activity of *Mbo* RNAP on the native *Mtb* AP3 promoter (more than an order of magnitude less than *Eco* RNAP, Figure 2A). This finding was echoed by the absence of a robust Dnase I footprint for *Mbo* RNAP on AP3 (Figure 5A, lane 6) and the absence of a KMnO_4_ footprint for *Mbo* RNAP on AP3 (Figure 6A, lane 6). The weak transcription activity of *Mbo* RNAP holoenzyme on AP3 is a property of the core RNAP and not *Mbo* σ^A^ (Figure 4C).
- High sensitivity of the *Mbo* RNAP to Cl^−^, particularly on AP3 (1/2-maximal activity on AP3 at about 60 mM KCl, compared to 160 mM KCl for *Eco* RNAP, Figure 2A).
- Very unstable *Mbo* RNAP open promoter complexes on both promoters (t_1/2_ of 2.3 min on AP3 and less than 1 min on AC50), while *Eco* RNAP formed essentially irreversible promoter complexes on both promoters (Figure 3). This finding was echoed in the rapid disappearance of the Dnase I and KMnO_4_ footprints for *Mbo* RNAP upon competitor challenge (Figure 5B, lanes 8 and 9; Figure 6F, lanes 2, 4, 6, 8, 10).

These significant differences between the *Mbo* and *Eco* RNAPs were, to a significant extent, rescued by the presence of the transcription activator CarD:

- The weak *Mbo* RNAP transcription activity on AP3 was boosted by CarD nearly 10-fold (Figure 2A).
- The very unstable promoter complexes formed by *Mbo* RNAP on both promoters were dramatically stabilized by CarD (t_1/2_ increased more than 10-fold for AP3, more than 60-fold for AC50; Figure 3). This was also reflected in the behavior of the Dnase I and KMnO_4_ footprints (Figures 5 and 6).

Remarkably, despite these profound effects of CarD on the stability of the *Mbo* RNAP promoter complexes, the presence of CarD stimulated the strength of the DNase I and KMnO_4_ footprints on the AC50 promoter (where the footprints in the absence of CarD could be compared) as expected, but did not alter the structure of the footprints (Figure 5B, compare lanes 6 and 7; Figure 6F, compare lanes 2 and 3). On the AP3 promoter, a mutant of CarD that altered the effectiveness of CarD as a transcription activator (CarD^W85A^) also did not alter the structure of the footprints (Figure 5A, lane 10; Figure 6B, lane 14).

The *in vitro* transcription conditions used here, which included a large excess of RNAP over promoter DNA and therefore favored RPo formation and transcription activity, were chosen to allow detection of the very weak transcription activity of *Mbo* RNAP on AP3. Such conditions explain how the *Mbo* RNAP promoter complexes on the optimized AC50 promoter can have such a rapid dissociation rate (short t_1/2_) but still show transcription levels similar to *Eco* RNAP (Figure 2B). Even in these strongly favorable conditions, however, AP3 transcription by *Mbo* RNAP without CarD was very weak (Figure 2A). These observations suggest that *Mbo* RNAP has a very fast on-rate on AC50, while on AP3 the on-rate is slow. On AC50, at high RNAP concentration favoring the formation of RPo, transcription activity is high (in the absence of competitor promoter trap DNA) despite the high off-rate, even in the absence of CarD (Figure 3B). Eliminating the forward reaction by adding competitor promoter trap DNA reveals the rapid dissociation from AC50 and the effect of CarD in stabilizing the AC50 RPo to dissociation (Figure 3B). AP3 activity, on the other hand, may be limited by a slow on-rate: the slow on-rate combined with the fast off-rate in the absence of CarD would yield very low levels of RPo and thus weak transcription activity. CarD stimulates AP3 activity by dramatically slowing the off-rate (Figure 3A), allowing the build-up of increased levels of RPo (we cannot rule out that CarD also affects the on-rate).

The most striking distinction between the AC50 and AP3 promoters is that AC50 harbors an optimal 17-bp −10/−35 spacer, while AP3 harbors a suboptimal 18-bp spacer (Figure 1). Nevertheless, the promoter spacer mutagenesis and swapping experiments demonstrate that the suboptimal −10/−35 spacer of AP3 is not the source of its inability to support transcription by the *Mbo* RNAP (Figure 4). *Mbo* RNAP transcription activity on several different mutant AP3 promoters with optimal 17-bp −10/−35 spacers [AP3Δ23 and AP3(AC50sp), Figure 4A] was just as weak as the wild-type AP3 promoter (Figure 4B). Thus, the characteristics of the AP3 promoter that give rise to such feeble *Mbo* RNAP transcription activity remain unknown. More extensive swapping of promoter regions, such as the discriminator (45,46), as well as targeted promoter mutagenesis, will be required to understand this property. Understanding the characteristics of the AP3 promoter that give rise to such weak *Mbo* RNAP transcription activity is important since it will shed light on mechanistic differences between the *Mbo* and *Eco* RNAPs, since *Eco* RNAP shows robust transcription activity on AP3.

Our current analysis allows us to extend the model for CarD function (19). The results establish that CarD dramatically stabilizes *Mbo* RNAP open promoter complexes (Figure 3) but does not alter the RNAP/promoter interactions as revealed by DNase I footprinting (Figure 5), and does not alter the transcription bubble as revealed by KMnO_4_ footprinting (Figure 6). Engineering a non-complementary transcription bubble in the AC50 promoter extended the half-life of the *Mbo* RPo to values similar to that of *Eco* and made the function of CarD redundant (Figures 3 and 7). These findings support a model whereby CarD forms favorable interactions with the upstream edge of the pre-formed transcription bubble, stabilizing the bubble against collapse. This implies that bubble collapse is a major contributor to the instability of *Mbo* RNAP promoter complexes. However, we do note that our findings neither support nor contradict models for CarD function that posit allosteric effects on RNAP/promoter interactions (47). We also note that the CarD mechanism may be more complex and could affect other steps in the pathway of RPo formation.

Depletion of CarD in *Msm* cells results in increased levels of 16S rRNA, leading to the initial proposal that CarD acts as a repressor of rRNA transcription (18). This was coupled to the finding that overexpression of CarD amino acids 1–104 could rescue the phenotype of ΔDksA in *Eco* cells, since DksA negatively regulates rRNA transcription *in vivo* (48). Our results here clearly confirm that CarD functions *in vitro* as a transcription activator, as was also shown previously (19). CarD is an essential global regulator in mycobacteria; it is found on the *Msm* chromosome at essentially all σ^A^ promoters (19). Its depletion leads to sensitivity to multiple cellular stresses (18). We suggest that depletion of CarD leads to indirect, pleiotropic effects that ultimately result in an increase of 16S rRNA levels (19).

The CarD/DNA interaction involves a universally conserved Trp (*Mbo* CarD^W85^) that is predicted to insert into the distorted minor groove at the upstream edge of the −10 element (19). The CarD^W85A^ substitution was previously shown to be defective in transcription activation (19). We show here that CarD^W85A^ is also defective in stabilizing *Mbo* RNAP promoter complexes. The t_1/2_ for CarD^W85A^ on AP3 is 71% of the wild-type CarD, while on AC50 it is only 21% (Figure 3). Nevertheless, CarD^W85A^ still retains significant capacity to stabilize *Mbo* RNAP promoter compexes, indicating that other CarD/promoter DNA interactions must contribute to CarD function. CarD^W85F^ exhibited transcription activation and promoter complex stabilization function similar to that of wild-type CarD, indicating that a bulky, hydrophobic (and likely aromatic) residue inserted into the upstream edge of the transcription bubble is sufficient for nearly full CarD function.

*Eco* has served as the archetypical organism on which the overwhelming majority of biochemical characterization of RNAP has been focused, and the properties of this RNAP have been assumed to be generally representative for all bacterial RNAPs. Our extensive comparisons of the properties of *Eco* and *Mbo* RNAPs at the same *in vitro* transcription conditions and on the same promoters demonstrate that some of these assumptions are not valid. Dnase I footprinting studies indicate that physical RNAP/promoter interactions are very similar between the two RNAPs, and KMnO_4_ footprinting indicates that the transcription bubble formed by both RNAPs at promoters is essentially identical. However, *Eco* RNAP is characterized by very stable open promoter complexes when double-stranded DNA is used as a competitor, with half-lives ranging between ∼10 and 100s of minutes. Even the *Eco rrn*B P1 promoter, the regulation of which is dependent on the instability of its open complexes (49), has a reported half-life of 20–58 min when double-stranded promoter DNA is used as a competitor (50,51). In particular, for promoters that are close to optimum (close to consensus −10 and −35 elements, optimal 17-bp −10/−35 spacer), *Eco* RNAP typically forms RPo irreversibly (52). This is borne out in our study, where *Eco* RNAP forms essentially irreversible open promoter complexes on both the optimized AC50 promoter and even on the suboptimal AP3 promoter (Figure 3). This is decidedly not the case for *Mbo* RNAP, which forms exceedingly short-lived promoter complexes on both AP3 and even on the optimized AC50 promoter (Figure 3). Thus, the assumption of RPo irreversibility for *Mbo* RNAP, even on optimal promoters, is not valid. Investigations of other RNAPs from non-*Eco* sources, such as *B. subtilis* (14), *Tth* (8), *T. aquaticus* (12) and *M. smegmatis* (15), have also noted characteristically unstable promoter complexes compared with *Eco*. The possibility that the properties of *Eco* RNAP may not be representative of most bacterial RNAPs, but rather that these properties make *Eco* RNAP an outlier, needs to be considered. It is interesting to note that CarD is found in *Bacillus*, *Thermus* and *Mycobacterium* species, where purified RNAPs have been found to generate relatively unstable open promoter complexes, but not in *Eco*, where RNAP generally forms exceedingly stable open complexes (19). Our finding that the *Mbo* σ factor is not the source of the observed RPo instability (Figure 4C) suggests that the instability comes from a property of the core RNAP. In general, important structural features of the bacterial RNAPs are highly conserved, but RNAPs from different bacterial lineages can differ substantially due to the presence/absence of so-called lineage-specific insertions (35). For example, previous studies found that deletion of 188-residues inserted in the middle of the *Eco* β′ Trigger-Loop [β′In6 according to the nomenclature of (35); SI3 according to the nomenclature of (53)] decreased the stability of promoter complexes by 10-fold (53). In RNAPs from *Bacillus*, *Thermus* and *Mycobacterium* species (which form unstable open complexes), β′In6 is absent (35), suggesting a correlation.

ChIP-seq experiments in *M. smegmatis* established that CarD is a global regulator, being present at essentially all promoter regions in the genome, suggesting that CarD is not a promoter-specific regulator (19). We have shown here that unstable promoter complexes seem to be a general property of *Mbo* RNAP, even on an optimized promoter like AC50 (Figure 3B). This suggests that CarD may be thought of as a general transcription factor that functions to prevent transcription bubble collapse, helping to compensate for the otherwise rapidly dissociating RNAP/promoter complexes throughout the genome.

## SUPPLEMENTARY DATA

Supplementary Data are available at NAR Online.

SUPPLEMENTARY DATA
