# Supplementary material for: Mycobacterial RNA polymerase forms unstable open promoter complexes that are stabilized by CarD
Source: Nucleic Acids Res. 2014 Dec 15;43(1):433–45. doi: 10.1093/nar/gku1231 (PMC4288152; doi:10.1093/nar/gku1231)
Supplement: SUPPLEMENTARY DATA [file supp_43_1_433__index.html]

Mycobacterial RNA polymerase forms unstable open promoter complexes that are stabilized by CarD — SUPPLEMENTARY DATA 

# Mycobacterial RNA polymerase forms unstable open promoter complexes that are stabilized by CarD

## SUPPLEMENTARY DATA

**Files in this Data Supplement:**

- SUPPLEMENTARY DATA
