# Supplementary material for: Mycobacterial RNA polymerase forms unstable open promoter complexes that are stabilized by CarD
Source: Nucleic Acids Res. 2014 Dec 15;43(1):433–45. doi: 10.1093/nar/gku1231 (PMC4288152; doi:10.1093/nar/gku1231)
Supplement: SUPPLEMENTARY DATA [file supp_gku1231_nar-02630-v-2014-File009.pdf]

Supplementary Figure Legends

Figure S1. SDS polyacrylamide gel of final gel filtration (GE Healthcare SD200) step. Peak 1 eluted at the void volume (aggregated protein). Peak 2 eluted at a volume consistent with monomeric *Mbo* RNAP.

Figure S2. Full con bubble promoter trap used as competitor.

(*A*) Sequence of the full con bubble promoter trap. The promoter sequence is derived from the *fullcon* promoter [selected for optimal binding; (1)] but with an engineered noncomplementary transcription bubble (-11 to +1).

(*B*) Control experiments demonstrating the effectiveness of the full con bubble promoter trap as a competitor. For the left lane of each pair (+ competitor), the full con bubble promoter trap (2 μM) and the transcription template (either the AC50 or AP3 promoters as indicated, 10 nM) were added to RNAP holoenzyme (50 nM) and transcription substrates simultaneously, then incubated in an abortive initiation reaction for 10 min. No competitor (- competitor) was added to control reactions under identical conditions. The absence of transcription for the '+ competitor' trials indicates that all the RNAP binds the full con bubble promoter trap before binding and transcribing the promoter template, and does not dissociate over the time course of the experiment. Filter binding experiments (not shown) indicate that RNAP does not dissociate from the full con bubble promoter trap over the course of several hours. Assays were performed in 10 mM KGlu for the AP3 promoter and 150 mM KGlu for the AC50 promoter as described in materials and methods.

Figure S3. Double exponential fit for promoter AP3 promoter lifetime assay. (*Mbo* RNAP; ±CarD). Assays were performed as described in Figure 3. Transcription assays were performed in transcription buffer (see materials and methods) in 10 mM KGlu.

References

1. Gaal, T., Ross, W., Estrem, S.T., Nguyen, L.H., Burgess, R.R. and Gourse, R.L. (2001) Promoter recognition and discrimination by EsigmaS RNA polymerase. *Molecular Microbiology*, **42**, 939–954.

Davis_FigS1

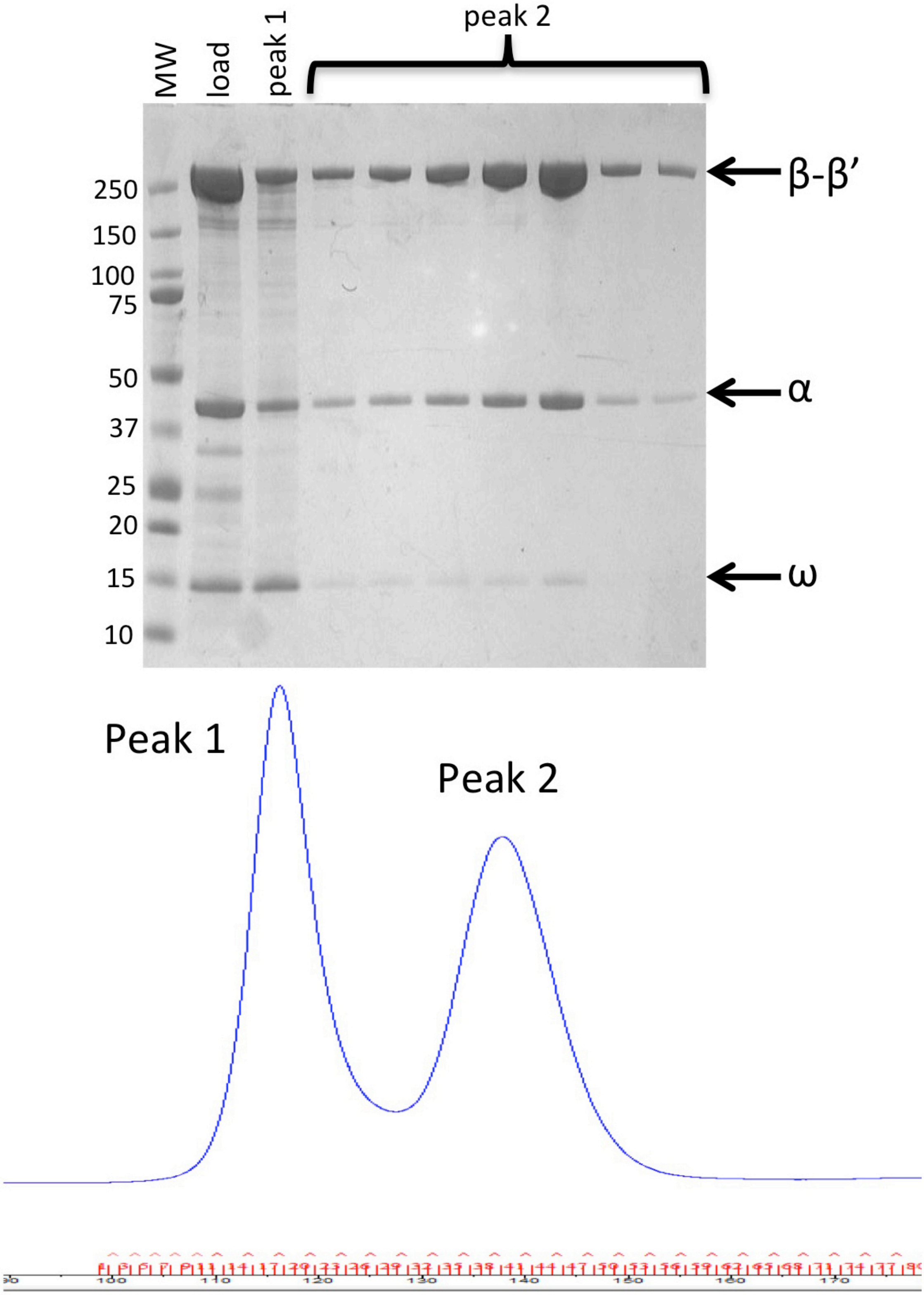

MW
load
peak 1
peak 2
250
150
100
75
50
37
25
20
15
10
β-β'
α
ω
Peak 1
Peak 2
100
110
120
130
140
150
160
170

Full con bubble promoter trap

A

```
     -35                                  -10
   element                              element
GGCTCTTGACAAAAGTGTTAAATTGTGCTATACTGGGATGGTATGGATGACAGAATTCGG
CCGAGAACTGTTTTCACAATTTAACACGA            ATACCTACTGTCTTAAGCC
                             ATACTGGGATGG
```

B

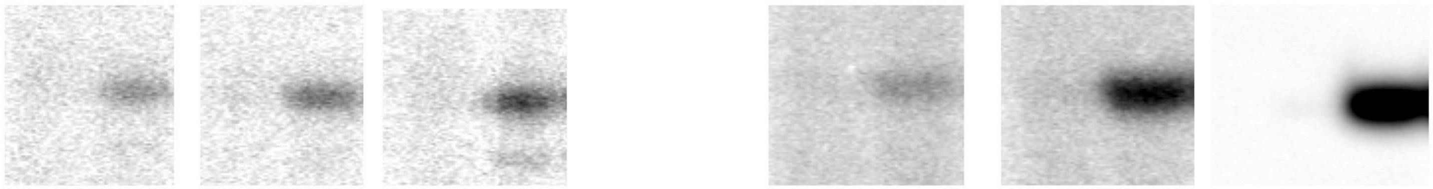

Davis et al, Figure S2

Davis_FigS3

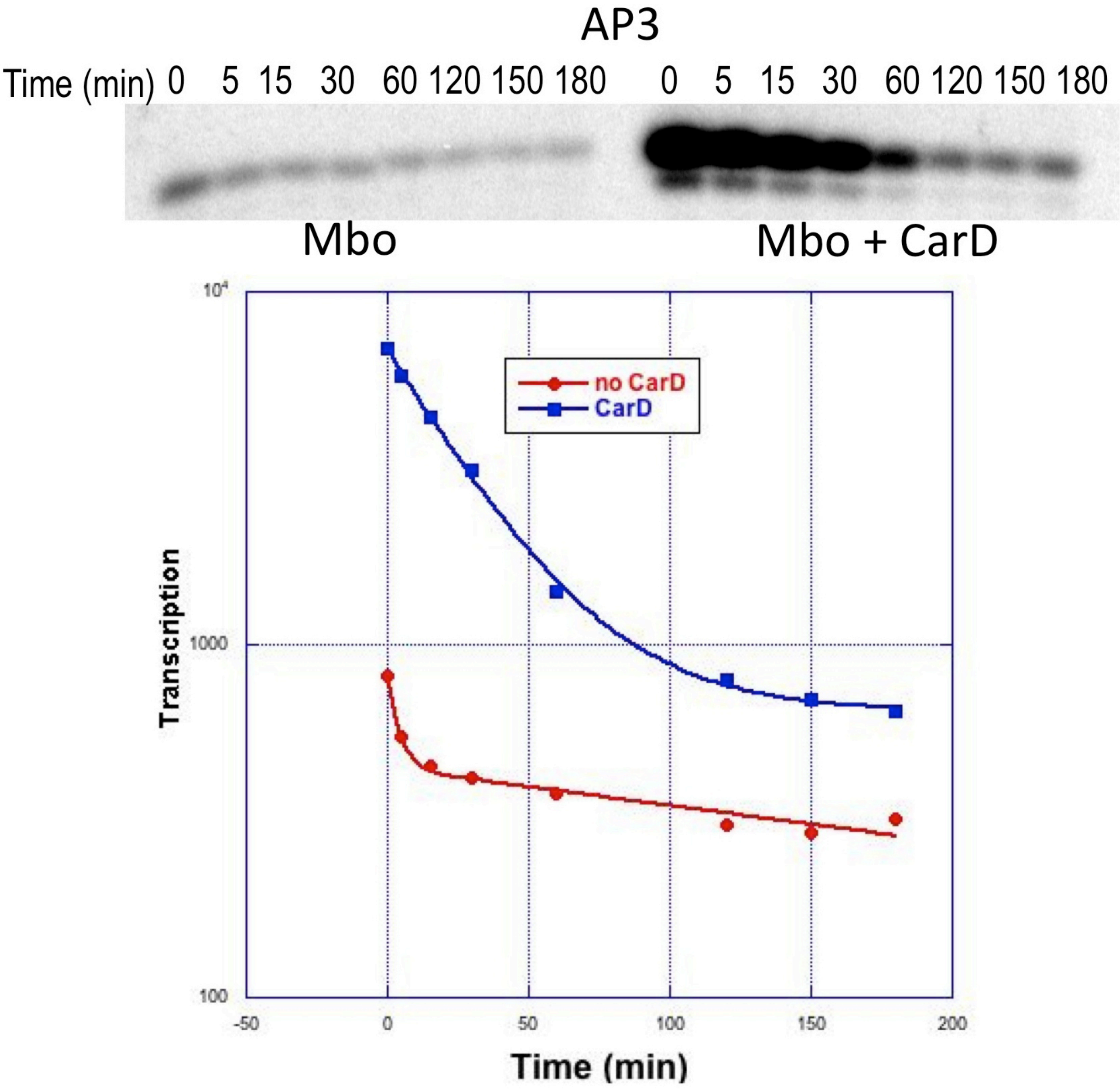

$$Y=C_1*\exp(-k_{off1})+C_2*\exp(-k_{off2})$$

C_1	372	6200
k_{off1}	0.299	0.0290
C_2	445	629
k_{off2}	0.0024	0.00013
